# Mediating effect analysis of visceral adiposity index on free triiodothyronine to free thyroxine ratio and non-alcoholic fatty liver disease in euthyroid population

**DOI:** 10.3389/fendo.2022.961803

**Published:** 2022-08-29

**Authors:** Huan-Xin Liu, Yan-Yan Ren, Cui-Qiao Meng, Zhong Li, Qian Nie, Chun-Hong Yu, Hui-Juan Ma

**Affiliations:** ^1^ Health Examination Center, Hebei General Hospital, Shijiazhuang, China; ^2^ Department of Neurology, Hebei General Hospital, Shijiazhuang, China; ^3^ Department of General Surgery, Shijiazhuang people’s Hospital, Shijiazhuang, China; ^4^ Key Laboratory of metabolic disease in Hebei Province, Hebei General Hospital, Shijiazhuang, China; ^5^ Department of Endocrinology, Hebei General Hospital, Shijiazhuang, China

**Keywords:** visceral adiposity index, free triiodothyronine to free thyroxine ratio, non-alcoholic fatty liver disease, thyroid function, mediating effect

## Abstract

**Background:**

The association between free triiodothyronine/free thyroxine (FT3/FT4) and non-alcoholic fatty liver disease (NAFLD) in euthyroid subjects is unclear. In addition, few studies have explored whether VAI mediates the association between FT3/FT4 ratio and NAFLD in the euthyroid population. We aimed to analyze the mediating effect of VAI on the FT3/FT4 ratio and NAFLD risk in the euthyroid population.

**Methods:**

This cross-sectional study included 7 946 annual health examinees from the Health Examination Center, Hebei General Hospital, from January to December 2020. The basic information and biochemical parameters, as well as calculated FT3/FT4 ratio and VAI were collected. NAFLD was diagnosed according to abdominal ultrasonography. The fibrosis score for NAFLD positive subjects (NFS) was calculated to reflect the extent of liver fibrosis. The risk of NAFLD was analyzed by quartiles of FT3/FT4 ratio (Q1-Q4 quartiles) and VAI (V1-V4 quartiles), respectively. Pearson correlation analysis was performed to investigate the correlation between FT3/FT4 ratio and VAI. Multivariate logistic regression analysis was applied to analyze the effect of FT3/FT4 ratio and VAI on NAFLD and NFS status. Bootstrap was conducted to explore whether VAI mediated the association between FT3/FT4 ratio and NAFLD.

**Results:**

Of the 7 946 participants, 2 810 (35.36%) had NAFLD and 5 136 (64.64%) did not. Pearson correlation analysis indicated that FT3/FT4 ratio was positively associated with VAI (*P*<0.05). Multivariate logistic regression analysis indicated that compared to the Q1 group, the risk of NAFLD significantly increased in Q3 group [OR=1.255, 95%CI (1.011, 1.559)] and Q4 group [OR=1.553, 95%CI (1.252, 1.926)](*P*<0.05). Compared to the V1 group, the risk of NAFLD notably increased in V2 group [OR=1.584, 95%CI (1.205, 2.083)], V3 group [OR=2.386, 95%CI (1.778, 3.202)] and V4 group [OR=4.104, 95%CI (2.835, 5.939)] (*P*<0.01). There was no relevance between FT3/FT4 ratio, VAI and NFS status. Mediating effect analysis showed that FT3/FT4 ratio significantly directly influenced NAFLD prevalence [β=3.7029, 95%CI (2.9583, 4.4474)], and VAI partly mediated the indirect effect of the FT3/FT4 ratio on NAFLD prevalence [β=2.7649, 95%CI (2.2347, 3.3466)], and the mediating effect accounted for 42.75% of the total effects.

**Conclusion:**

Both FT3/FT4 ratio and VAI were predictors of NAFLD, and VAI partly mediated the indirect effect of the FT3/FT4 ratio on NAFLD prevalence in the euthyroid population.

## Introduction

Non-alcoholic fatty liver disease (NAFLD) is a common chronic liver disease globally with a prevalence of about 25% (1). The disease spectrum of NAFLD includes simple fatty liver (SFL) and non-alcoholic steatohepatitis (NASH) (with or without hepatic fibrosis). SFL may transition to NASH under certain conditions. Not only does NAFLD increases the risk of liver fibrosis, liver cirrhosis and hepatocellular carcinoma, but it also is closely related to obesity, abnormal lipid metabolism and decreased insulin sensitivity, which leads to extrahepatic complications such as type 2 diabetes, cardiovascular diseases and cerebrovascular diseases, and so on (2–4). NAFLD has become a significant public health concern affecting human health. NAFLD is estimated to become the leading cause of end-stage liver disease in the following decades (5).

Studies have shown that thyroid hormones play an essential role in regulating lipid and carbohydrate metabolism, and thyroid dysfunction is closely related to various liver diseases (6–10). Studies have also indicated that fluctuation of thyroid hormones within the euthyroid range impacts atherosclerosis and biochemical markers of increased cardiovascular risk (9). Thyroid hormone levels stimulate the transportation of free fatty acids (FFA) to the liver for reesterification into triglyceride (TG), and affect fatty acid β-oxidation and lipid synthesis, thereby affecting hepatic fat accumulation and increasing the risks of NAFLD and liver fibrosis (10, 11). In addition, studies have reported that free triiodothyronine/free thyroxine (FT3/FT4) was evaluated as an indirect indicator of peripheral deiodinase activity, which reflects the peripheral sensitivity of thyroid hormones and predicts NAFLD better (3, 12). However, there are few related studies on the Chinese population (13).

As a crucial indicator of visceral obesity, the visceral adiposity index (VAI) reflects the distribution and accumulation of visceral fat (14). Studies have shown that VAI was a strong predictor of NAFLD risk (15). However, few studies have explored whether VAI mediated the association between FT3/FT4 ratio and NAFLD in euthyroid population. Therefore, this study aimed to investigate the association between FT3/FT4 ratio and NAFLD in euthyroid population. Simultaneously, the mediating effect of VAI on the association between FT3/FT4 ratio and NAFLD was analyzed to provide new ideas and more clinical evidence for early identification and intervention of NAFLD.

## Materials and methods

### Study participants

A total of 10,526 annual health examinees were collected from the Health Examination Center, Hebei General Hospital from January to December 2020. Inclusion criteria: (1) age ≥18 years old; (2) normal thyroid function. Exclusion criteria: (1) Previously or currently diagnosed with thyroid diseases or taken drugs that affected thyroid function (n=292). (2) Previously diagnosed with other liver diseases, such as viral liver disease, alcoholic liver disease, drug-induced liver disease, autoimmune liver disease, genetic metabolic liver disease, et al. (n=178). (3) Previously long-term heavy drinking or recently cumulative alcohol intake (≥140 g per week for men and ≥70 g per week for women (n=773). (4) Patients with malignant tumors, hypothalamus, pituitary disease, liver and kidney insufficiency (n=335). (5) Pregnant or lactating women (n=36). (6) Missing questionnaire or physical examination data (n=966). The results were based on the last physical examination for those with more than one examination during the period. After exclusion, the final retrospective cross-sectional analysis included 7,946 participants. All research subjects were informed consent, and the study protocol was approved by the Ethics Committee of the Hebei General Hospital.

### Data collection

The basic information was collected, including gender, age, past disease history, and current medication history. Anthropometric measurements were collected, including height, weight and waist circumference (WC). Height and weight were measured while the participants wore light clothes and no shoes. These measurements were made twice during the physical examination, and the mean values were applied to subsequent analyses. The body mass index (BMI) was calculated as body mass in kilograms divided by the square of height in meters. Systolic blood pressure (SBP) and diastolic blood pressure (DBP) were measured 3 times using an automated sphygmomanometer (OMRON, HEM-7125, Dalian, China). The participants rested for >5 min before measuring their blood pressure. The average of the three readings was used for each blood pressure parameter. After fasting for 8-12 hours, 5ml of peripheral venous blood samples were drawn and centrifuged at 3000 r/min with a radius of 10 cm for 15 minutes. The serum was preserved and processed routinely. The biochemical parameters were determined by Hitachi 7600-110 automatic biochemical analyzer, including thyroid-stimulating hormone (TSH), free triiodothyronine (FT3), free thyroxine (FT4), triiodothyronine (TT3), and tetraiodothyronine (TT4), platelet (PLT), aspartate aminotransferase (AST), alanine aminotransferase (ALT), gamma-glutamyltransferase (γ-GGT), albumin (ALB), fasting blood glucose (FPG), serum uric acid (SUA), serum creatinine (Scr), total cholesterol (TC), TG, low density lipoprotein cholesterol (LDL-C) and high density lipoprotein cholesterol (HDL-C). Peripheral sensitivity indices for thyroid hormones (FT3/FT4) were calculated based on the levels of FT3 and FT4.

### Definition of NAFLD

Clinical diagnostic criteria for NAFLD: (1) No history of alcohol intake or weekly alcohol intake<140g in males and<70g in females. (2) The liver imaging diagnosis was consistent with diffuse fatty liver after excluding other causes. Ultrasound imaging diagnosis: (1) The liver near-field echogenicity is enhanced diffusely (“bright liver”) and is stronger than that of the kidney; (2) The structure of the intrahepatic duct is unclear; (3) The liver far-field echogenicity decreased gradually. NAFLD was defined by at least two positive ultrasound findings above (16).

Among NAFLD positive subjects, to reflect the extent of liver fibrosis, the NAFLD fibrosis score (NFS) was calculated as NFS= −1.675+0.037×age (years)+0.094×BMI (kg/m^2^)+1.13× impaired FPG/presence of diabetes (yes=1, no=0)+0.99×AST/ALT ratio-0.013×PLT count (×10^9^/L) -0.66×ALB (g/dL). Subjects was further categorised into two groups according to NFS levels: low NFS group (NFS<-1.455) and high NFS group (NFS≥-1.455) (17).

### Normal thyroid function range

TSH: 0.560-5.910 μU/ml, FT3: 3.28-6.47 pmol/L, FT4: 7.64-16.03 pmol/L, TT3: 1.01-2.48 nmol/L, TT4: 69.97-152.52 nmol/L.

### VAI calculation formula


Male:VAI=WC(cm)/(39.68+1.88×BMI)×TG/1.03×1.31/HDL−C;   Female: VAI=WC(cm)/(36.58+1.89×BMI)×TG/0.81×1.52/HDL−C.

### Stratification at FT3/FT4 ratio and VAI levels

FT3/FT4 ratio was divided into Q1~Q4 groups according to quartiles: Q1 group ≤ 4.305 (n=2212), 4.305<Q2 group ≤ 4.997 (n=1926), 4.997<Q3 group ≤ 5.786(n=1959), Q4 Group>5.786 (n=1849).

VAI was divided into V1~V4 groups according to quartiles: V1 group ≤ 0.959 (n=1987), 0.959<V2 group ≤ 1.465 (n=1986), 1.465<V3 group ≤ 2.285 (n=1986), V4 group>2.285 (n=1987).

### Statistical analysis

SPSS 22.0 statistical software was used for data analysis. Continuous variables with normal distribution were expressed as means ± standard deviation ( $\bar{x}$ ± s), and One-way ANOVA analysis was performed to compare multiple groups. Independent t-test was taken to compare multiple groups. While continuous variables with skewed distribution were shown as medians (interquartile ranges), the Kruskal-Wallis rank-sum test was conducted to compare multiple groups. All categorical variables were expressed as relative numbers, the groups were compared using χ^2^ test. Pearson correlation analysis was performed to investigate the correlation between FT3/FT4 ratio, VAI and various risk factors of NAFLD. The effects of FT3/FT4 ratio and VAI on NAFLD and NFS status were analyzed by multivariate logistic regression. Bootstrap was conducted to explore whether VAI mediated the association between FT3/FT4 ratio and NAFLD. *P-values<*0.05 were considered statistically significant.

## Results

**Figure 1:**
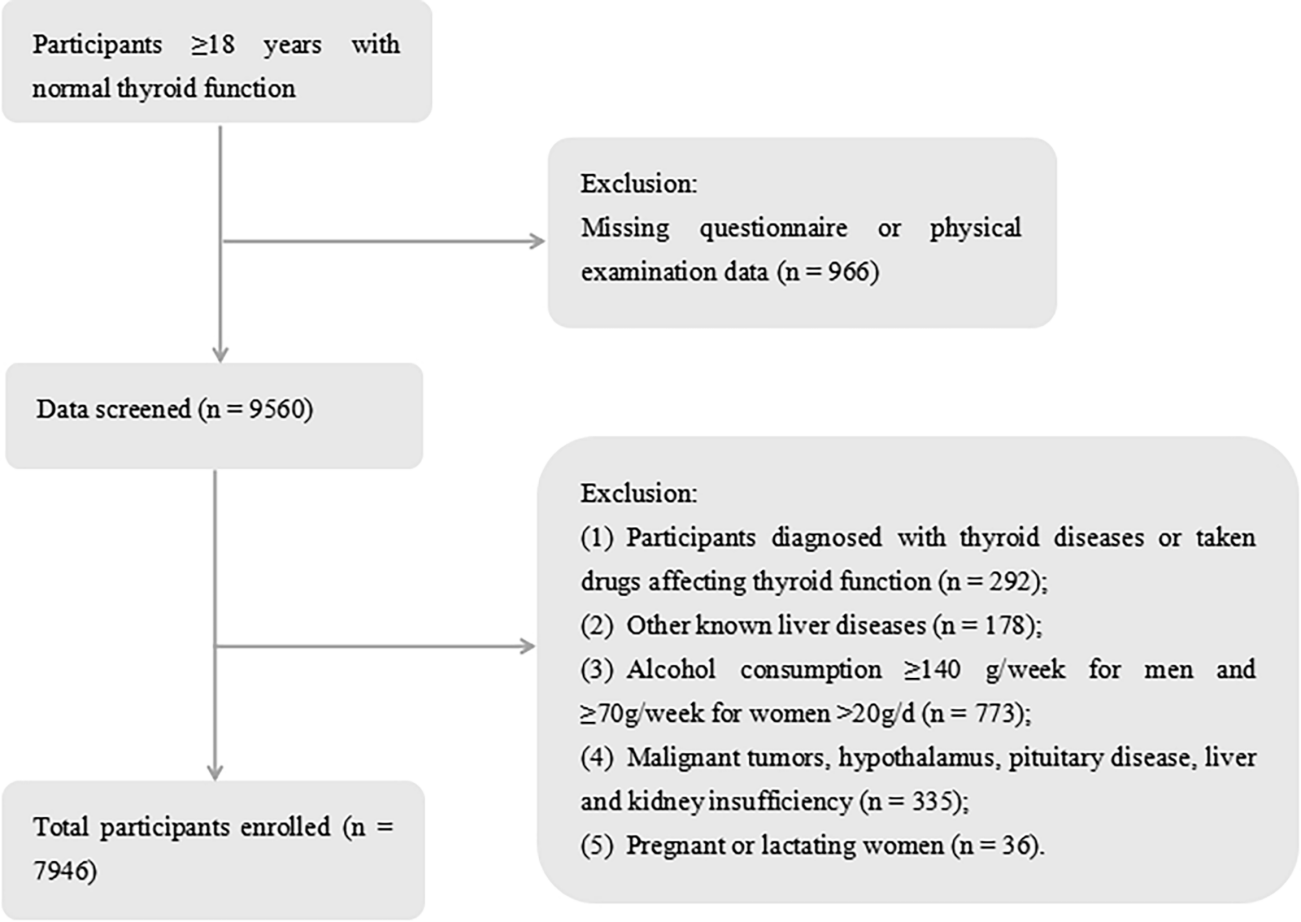
Flowchart of the inclusion and exclusion of participants.

A total of 10,526 participants were registered in this study from January to December 2020. After excluding 2,580 participants because of exclusion criteria, the final study group consisted of 7,946 euthyroid participants, including 3,632 (45.71%) males and 4,314 (54.29%) females (**Figure 1**).

### Clinical characteristics of the participants

A total of 7,946 participants ranged from 18 to 88 years old in the final analysis. The average age was 47.64 ± 13.21 years old. The incidence of NAFLD was 35.36% (2810/7946). The age, sex, WC, BMI, SBP, DBP, TC, TG, LDL-C, HDL-C, SUA, Scr, AST, ALT, AST/ALT, γ-GGT, TSH, VAI and NAFLD prevalence were significantly different in the Q1~Q4 groups *(P*<0.05). The difference in FPG between the four groups was not statistically significant (*P*>0.05) (**Table 1**). The levels of age, sex, WC, BMI, SBP, DBP, TC, TG, LDL-C, HDL-C, SUA, Scr, AST, ALT, AST/ALT, γ-GGT, FT3, FT4, FT3/FT4, TSH and VAI were significantly different in the V1~V4 groups. Moreover, the prevalence of NAFLD increased with elevated VAI levels (*P*<0.05) (**Table 2**).

**Table 1:** Comparison of basic demographic characteristics of physical examinees with normal thyroid function in quartile groups of FT3/FT4 ratio.

	Q1 (n=2212)	Q2 (n=1926)	Q3 (n=1959)	Q4 (n=1849)	F/χ²	*P*
Gender					17.317	0.001
Male〔n(%)〕	947(42.81)	908(47.14)	874(44.61)	903(48.84)		
Female〔n(%)〕	1265(57.19)	1018(52.86)	1085(55.39)	946(51.16)		
Age(years)	47.64 ± 13.21	46.98 ± 12.28	46.63 ± 11.62	46.68 ± 11.52	3.028	0.028
WC(cm)	83.97 ± 11.57	85.82 ± 11.45	86.23 ± 11.55	88.06 ± 11.36	43.175	<0.001
BMI(kg/m^2^)	23.86 ± 3.58	24.44 ± 3.51	24.65 ± 3.64	25.26 ± 3.53	53.183	<0.001
SBP(mmHg)	120.03 ± 18.57	121.43 ± 17.59	121.8 ± 17.92	123.58 ± 17.38	13.336	<0.001
DBP(mmHg)	79.06 ± 11.09	80.55 ± 11.17	80.82 ± 11.41	82.40 ± 11.55	29.68	<0.001
TC(mmol/L)	5.00 ± 0.98	5.04 ± 0.93	5.15 ± 0.97	5.21 ± 0.96	20.704	<0.001
TG(mmol/L)	1.30 ± 0.94	1.48 ± 1.15	1.57 ± 1.37	1.82 ± 1.41	309.035	<0.001
LDL-C(mmol/L)	3.13 ± 0.73	3.17 ± 0.70	3.26 ± 0.73	3.30 ± 0.71	24.097	<0.001
HDL-C(mmol/L)	1.40 ± 0.30	1.37 ± 0.29	1.37 ± 0.30	1.34 ± 0.29	12.511	<0.001
FPG(mmol/L)	5.72 ± 1.70	5.65 ± 1.39	5.67 ± 1.29	5.75 ± 1.31	60.373	0.192
SUA(μmol/L)	323.68 ± 87.82	335.19 ± 88.84	336.55 ± 91.73	347.62 ± 95.99	23.434	<0.001
Scr(μmol/L)	66.66 ± 12.63	66.36 ± 12.59	65.36 ± 12.57	65.13 ± 12.94	6.885	<0.001
AST(U/L)	20.98 ± 8.54	21.65 ± 10.03	22.66 ± 10.52	23.81 ± 13.82	26.011	<0.001
ALT(U/L)	18.83 ± 12.97	21.01 ± 18.17	23.21 ± 17.74	25.62 ± 20.34	56.840	<0.001
AST/ALT	1.30 ± 0.46	1.23 ± 0.44	1.18 ± 0.46	1.13 ± 0.48	50.118	<0.001
γ-GGT (U/L)	26.95 ± 29.19	29.93 ± 31.99	31.93 ± 29.12	36.32 ± 32.43	20.412	<0.001
TSH(μIU/mL)	2.10 ± 0.99	2.20 ± 1.05	2.27 ± 1.09	2.40 ± 1.16	66.906	<0.001
VAI	1.61 ± 1.37	1.88 ± 1.85	2.01 ± 2.15	2.33 ± 2.13	277.767	<0.001
NAFLD[n(%)]	577(26.08)	630(32.71)	732(37.37)	871(47.11)	204.229	<0.001

WC, waist circumference; BMI, body mass index; SBP, systolic blood pressure; DBP, diastolic blood pressure; TC, total cholesterol; TG, triglyceride; LDL-C, low density lipoprotein cholesterol; HDL-C, high density lipoprotein cholesterol; FPG, fasting plasma glucose; SUA, serum uric acid; Scr, serum creatinine; AST, aspartate aminotransferase; ALT, alanine aminotransferase; AST/ALT, aspartate aminotransferase/alanine aminotransferase; γ-GGT, gamma-glutamyltransferase; TSH, thyroid-stimulating hormone; VAI, visceral adiposity index; NAFLD, non-alcoholic fatty liver disease. Data are means ± standard deviations or medians (interquartile ranges) for continuous variables, and numbers (proportions) for categorical variables. P values are calculated by One-way ANOVA analysis and Kruskal-Wallis rank-sum test for continuous variables, Chi-square test for categorical variables. P<0.05 was considered statistically significant.

**Table 2:** Comparison of basic demographic characteristics of physical examinees with normal thyroid function in quartile groups of visceral adiposity index.

	V1 (n=1987)	V2 (n=1986)	V3 (n=1986)	V4 (n=1987)	F/χ²	*P*
Gender					185.094	<0.001
Male[n(%)]	703(35.38)	832(41.89)	1004(50.55)	1093(55.01)		
Female[n(%)]	1284(64.62)	1154(58.11)	982(49.45)	894(44.99)		
Age(years)	43.17 ± 12.59	46.13 ± 11.95	49.34 ± 11.95	49.39 ± 11.28	123.521	<0.001
WC(cm)	78.42 ± 10.17	84.12 ± 10.50	88.72 ± 10.39	92.45 ± 10.24	683.419	<0.001
BMI(kg/m^2^)	22.34 ± 3.08	23.97 ± 3.30	25.33 ± 3.30	26.45 ± 3.35	588.826	<0.001
SBP(mmHg)	114.99 ± 16.37	119.67 ± 17.71	124.40 ± 17.72	127.47 ± 17.41	198.398	<0.001
DBP(mmHg)	76.2 ± 10.17	79.53 ± 11.22	82.25 ± 11.15	84.55 ± 11.11	215.398	<0.001
TC(mmol/L)	4.84 ± 0.89	5.01 ± 0.92	5.22 ± 0.99	5.30 ± 0.97	97.601	<0.001
TG(mmol/L)	0.71 ± 0.17	1.05 ± 0.22	1.50 ± 0.33	2.87 ± 1.80	2084.885	<0.001
LDL-C(mmol/L)	2.90 ± 0.65	3.16 ± 0.69	3.39 ± 0.74	3.40 ± 0.68	227.331	<0.001
HDL-C(mmol/L)	1.62 ± 0.29	1.41 ± 0.23	1.30 ± 0.23	1.16 ± 0.21	1297.672	<0.001
FPG(mmol/L)	5.27 ± 0.94	5.51 ± 1.16	5.85 ± 1.47	6.16 ± 1.87	151.816	<0.001
SUA(μmol/L)	298.49 ± 76.97	319.57 ± 82.46	346.69 ± 88.91	376.12 ± 96.84	299.97	<0.001
Scr(μmol/L)	63.34 ± 11.59	65.50 ± 12.19	66.92 ± 12.73	67.87 ± 13.71	48.644	<0.001
AST(U/L)	20.56 ± 7.76	21.35 ± 10.59	22.55 ± 11.15	24.40 ± 12.84	47.914	<0.001
ALT(U/L)	17.02 ± 11.63	19.93 ± 15.43	23.52 ± 19.68	27.59 ± 20.13	142.093	<0.001
AST/ALT	1.40 ± 0.48	1.26 ± 0.45	1.14 ± 0.43	1.04 ± 0.41	240.706	<0.001
γ-GGT(U/L)	21.49 ± 19.43	25.68 ± 22.03	33.36 ± 30.27	42.52 ± 40.73	121.023	<0.001
FT3(pmol/L)	4.98 ± 0.50	5.05 ± 0.50	5.15 ± 0.50	5.23 ± 0.52	92.067	<0.001
FT4(pmol/L)	11.71 ± 1.49	11.58 ± 1.45	11.54 ± 1.49	11.40 ± 1.52	14.531	<0.001
FT3/FT4	0.43 ± 0.07	0.44 ± 0.07	0.45 ± 0.07	0.47 ± 0.07	92.575	<0.001
TSH(μIU/mL)	2.12 ± 1.02	2.26 ± 1.10	2.24 ± 1.05	2.34 ± 1.12	14.138	<0.001
NAFLD[n(%)]	174(8.76)	467(23.51)	855(43.05)	1314(66.13)	1611.553	<0.001

WC, waist circumference; BMI, body mass index; SBP, systolic blood pressure; DBP, diastolic blood pressure; TC, total cholesterol; TG, triglyceride; LDL-C, low density lipoprotein cholesterol; HDL-C, high density lipoprotein cholesterol; FPG, fasting plasma glucose; SUA, serum uric acid; Scr, serum creatinine; AST, aspartate aminotransferase; ALT, alanine aminotransferase; AST/ALT, aspartate aminotransferase/alanine aminotransferase; γ-GGT, gamma-glutamyltransferase; FT3, free triiodothyronine; FT4, free thyroxine; FT3/FT4, free triiodothyronine/free thyroxine; TSH, thyroid-stimulating hormone; NAFLD, non-alcoholic fatty liver disease. Data are means ± standard deviations or medians (interquartile ranges) for continuous variables, and numbers (proportions) for categorical variables. P values are calculated by One-way ANOVA analysis and Kruskal-Wallis rank-sum test for continuous variables, Chi-square test for categorical variables. P<0.05 was considered statistically significant.

### Comparison of basic demographic characteristics of NAFLD positive participants with low NFS and High NFS

NAFLD positive participants with high NFS were relatively older and more likely to be male, had higher WC, BMI, SBP, DBP, FPG, AST/ALT and FT4, and lower TC, LDL-C, HDL-C, SUA, ALT, FT3 and FT3/FT4 when compared to their low NFS participants (*P*<0.05). Compared to the low NFS group, the levels of TG, Scr, AST, γ-GGT, TSH and VAI had no difference in the high NFS group (P>0.05) (**Table 3**).

**Table 3:** Comparison of basic demographic characteristics of NAFLD positive subjects with low NFS and high NFS.

	NAFLD with low NFS (n=1983)	NAFLD with high NFS (n=827)	t/χ²	*P*
Gender			4.967	0.026
Male[n(%)]	1280 (64.55)	570(68.92)		
Female[n(%)]	70 3(35.45)	257(31.08)		
Age(years)	46.63 ± 9.64	58.53 ± 10.04	-29.451	<0.001
WC(cm)	93.05 ± 9.06	97.52 ± 9.93	-11.595	<0.001
BMI(kg/m^2^)	26.77 ± 3.02	27.95 ± 3.53	-8.972	<0.001
SBP(mmHg)	126.70 ± 15.95	135.32 ± 17.24	-12.755	<0.001
DBP(mmHg)	85.38 ± 11.10	86.76 ± 10.70	-3.041	0.002
TC(mmol/L)	5.35 ± 0.96	5.12 ± 1.04	5.687	<0.001
TG(mmol/L)	2.13 ± 1.53	2.14 ± 1.61	-0.152	0.879
LDL-C(mmol/L)	3.46 ± 0.70	3.27 ± 0.76	6.324	<0.001
HDL-C(mmol/L)	1.25 ± 0.24	1.22 ± 0.24	2.599	0.009
FPG(mmol/L)	5.79 ± 1.30	7.23 ± 2.20	-21.518	<0.001
SUA(μmol/L)	387.75 ± 90.84	367.46 ± 85.52	5.489	<0.001
Scr(μmol/L)	69.32 ± 13.24	69.84 ± 12.64	-0.954	0.340
AST(U/L)	25.02 ± 12.01	25.60 ± 16.30	-1.032	0.302
ALT(U/L)	31.40 ± 22.01	27.00 ± 20.90	4.900	<0.001
AST/ALT	0.92 ± 0.29	1.10 ± 0.46	-12.706	<0.001
γ-GGT (U/L)	42.85 ± 37.54	42.82 ± 41.60	0.016	0.987
FT3(pmol/L)	5.30 ± 0.49	5.14 ± 0.50	7.918	<0.001
FT4(pmol/L)	11.45 ± 1.46	11.61 ± 1.56	-2.575	0.01
FT3/FT4	0.47 ± 0.07	0.45 ± 0.07	6.744	<0.001
TSH(μIU/mL)	2.22 ± 1.04	2.25 ± 1.06	-0.839	0.402
VAI	2.77 ± 2.39	2.90 ± 2.60	-1.269	0.204

WC, waist circumference; BMI, body mass index; SBP, systolic blood pressure; DBP, diastolic blood pressure; TC, total cholesterol; TG, triglyceride; LDL-C, low density lipoprotein cholesterol; HDL-C, high density lipoprotein cholesterol; FPG, fasting plasma glucose; SUA, serum uric acid; Scr, serum creatinine; AST, aspartate aminotransferase; ALT, alanine aminotransferase; AST/ALT, aspartate aminotransferase/alanine aminotransferase; γ-GGT, gamma-glutamyltransferase; FT3, free triiodothyronine; FT4, free thyroxine; FT3/FT4, free triiodothyronine/free thyroxine; TSH, thyroid-stimulating hormone; VAI, visceral adiposity index. Data are means ± standard deviations or medians (interquartile ranges) for continuous variables, and numbers (proportions) for categorical variables. P values are calculated by independent samples t-test or nonparametric rank-sum test for continuous variables, Chi-square test for categorical variables. P<0.05 was considered statistically significant.

### Pearson correlation analysis of FT3/FT4 ratio, visceral adiposity index and basic indicators

Pearson correlation analysis indicated that FT3/FT4 ratio was positively correlated with WC (r=0.133, *P*<0.001), BMI (r=0.150, *P*<0.001), SBP (r=0.071, *P*<0.001), DBP (r=0.109, *P*<0.001), TC(r=0.095, *P*<0.001), TG (r=0.163, *P*<0.001), LDL-C (r=0.101, *P*<0.001), SUA (r=0.101, *P*<0.001), AST (r=0.110, *P*<0.001), ALT (r=0.158, *P*<0.001), γ-GGT (r=0.119, *P*<0.001), TSH (r=0.099, *P*<0.001) and VAI (r=0.147, *P*<0.001) but negatively correlated with age (r=-0.041, *P*<0.001), HDL-C (r=-0.066, *P*<0.001), Scr (r=-0.047, *P*<0.001) and AST/ALT (r=-0.140, *P*<0.001). Similarly, VAI was positively correlated with age (r=0.108, *P*<0.001), WC (r=0.315, *P*<0.001), BMI (r=0.294, *P*<0.001), SBP (r=0.194, *P*<0.001), DBP (r=0.215, *P*<0.001), TC (r=0.159, *P*<0.001), TG (r=0.947, *P*<0.001), LDL-C (r=0.136, *P*<0.001), FPG (r=0.231, *P*<0.001), SUA (r=0.253, *P*<0.001), Scr (r=0.087, *P*<0.001), AST (r=0.116, *P*<0.001), ALT (r=0.174, *P*<0.001), γ-GGT (r=0.222, *P*<0.001) and TSH (r=0.051, *P*<0.001) but negatively correlated with HDL-C (r=-0.438, *P*<0.001) and AST/ALT (r=-0.188, *P*<0.001) (**Table 4**).

**Table 4:** Pearson correlation analysis of FT3/FT4 ratio, visceral adiposity index and basic physical and biochemical indicators.

	FT3/FT4		VAI	
	r	*P*	r	*P*
Age	-0.041	<0.001	0.108	<0.001
WC	0.133	<0.001	0.315	<0.001
BMI	0.150	<0.001	0.294	<0.001
SBP	0.071	<0.001	0.194	<0.001
DBP	0.109	<0.001	0.215	<0.001
TC	0.095	<0.001	0.159	<0.001
TG	0.163	<0.001	0.947	<0.001
LDL-C	0.101	<0.001	0.136	<0.001
HDL-C	-0.066	<0.001	-0.438	<0.001
FPG	0.007	0.504	0.231	<0.001
SUA	0.101	<0.001	0.253	<0.001
Scr	-0.047	<0.001	0.087	<0.001
AST	0.110	<0.001	0.116	<0.001
ALT	0.158	<0.001	0.174	<0.001
AST/ALT	-0.140	<0.001	-0.188	<0.001
γ-GGT	0.119	<0.001	0.222	<0.001
TSH	0.099	<0.001	0.051	<0.001
VAI	0.147	<0.001	—	—

WC, waist circumference; BMI, body mass index; SBP, systolic blood pressure; DBP, diastolic blood pressure; TC, total cholesterol; TG, triglyceride; LDL-C, low density lipoprotein cholesterol; HDL-C, high density lipoprotein cholesterol; FPG, fasting plasma glucose; SUA, serum uric acid; Scr, serum creatinine; AST, aspartate aminotransferase; ALT, alanine aminotransferase; AST/ALT, aspartate aminotransferase/alanine aminotransferase; γ-GGT, gamma-glutamyltransferase; TSH, thyroid-stimulating hormone; VAI, visceral adiposity index. P<0.05 were considered statistically significant.

### Multivariate logistic regression analysis of the association between FT3/FT4 ratio and the risk of NAFLD

FT3/FT4 ratio as an independent variable was assigned with NAFLD as a dependent variable (assignment: yes=1, no=0). Multivariate logistic analysis indicated that FT3/FT4 ratio was positively associated with NAFLD prevalence without adjusting confounding factors (*P*<0.001) (Model 1). Compared to the Q1 group, the risk of NAFLD significantly increased in Q2 group〔OR=1.400, 95%CI (1.214, 1.615)〕, Q3 group 〔OR=1.848, 95%CI (1.605, 2.127)〕 and Q4 group 〔OR=2.748, 95%CI (2.387, 3.164)〕 after adjustment for age and gender (*P*<0.001) (Model 2). After further adjustment for WC, BMI, SBP, DBP, TC, TG, LDL-C, HDL-C, FPG, SUA, Scr, AST, ALT, γ-GGT and TSH, the risk of NAFLD still significantly increased in Q3 group 〔OR=1.255, 95%CI (1.011, 1.559)〕 and Q4 group 〔OR=1.553, 95%CI (1.252, 1.926)〕 comparing with Q1 group (*P*<0.05) (Model 3) (**Table 5**).

**Table 5:** Multivariate logistic regression analysis of the association between FT3/FT4 ratio and the risk of NAFLD.

FT3/FT4	Model 1		Model 2		Model 3	
	OR(95%*CI*)	*P*	OR(95%*CI*)	*P*	OR(95%*CI*)	*P*
Q1	1.000	—	1.000	—	1.000	—
Q2	1.377 (1.2041.214∼1.576)	<0.001	1.400 (1.214∼1.615)	<0.001	1.030 (0.829∼1.279)	0.789
Q3	1.690 (1.482∼1.929)	<0.001	1.848 (1.605∼2.127)	<0.001	1.255 (1.011∼1.559)	0.039
Q4	2.524 (2.212∼2.879)	<0.001	2.748 (2.387∼3.164)	<0.001	1.553 (1.252∼1.926)	<0.001

Model 1 was not adjusted for confounding factors. Model 2 was adjusted for age and gender. Model 3 was adjusted for the covariates of model 2 plus waist circumference, body mass index, systolic blood pressure, diastolic blood pressure, total cholesterol, triglyceride, low density lipoprotein cholesterol, high density lipoprotein cholesterol, fasting plasma glucose, serum uric acid, serum creatinine, aspartate aminotransferase, alanine aminotransferase, gamma-glutamyltransferasethyroid, thyroid-stimulating hormone. Odds ratios and 95% CIs were calculated per 1-SD increment of FT3/FT4. FT3/FT4, free triiodothyronine to free thyroxine ratio; OR, odds ratio; CI, confidence interval.

### Multivariate logistic regression analysis of the association between VAI and the risk of NAFLD

The level of VAI as an independent variable was assigned with NAFLD as a dependent variable (assignment: yes=1, no=0). Multivariate logistic analysis indicated that VAI was positively associated with NAFLD prevalence without adjusting confounding factors (*P*<0.001) (Model 1). Compared to the V1 group, the risk of NAFLD significantly increased in V2 group 〔OR=3.070, 95%CI (2.531, 3.724)〕, V3 group 〔OR=6.977, 95%CI (5.797, 8.398)〕 and V4 group 〔OR=18.668, 95%CI (15.474, 22.523)〕 after adjustment for age and gender (*P*<0.001) (Model 2). After further adjustment for WC, BMI, SBP, DBP, TC, TG, LDL-C, HDL-C, FPG, SUA, Scr, AST, ALT, γ-GGT and TSH, the risk of NAFLD still significantly increased in V2 group 〔OR=1.584, 95%CI (1.205, 2.083)〕, V3 group 〔OR=2.386, 95%CI (1.778, 3.202)〕 and V4 group 〔OR=4.104, 95%CI (2.835, 5.939)〕 comparing with V1 group (*P*<0.01) (Model 3) (**Table 6**).

**Table 6:** Multivariate logistic regression analysis of the association between VAI and the risk of NAFLD.

VAI	Model 1		Model 2		Model 3	
	OR(95%CI)	*P*	OR(95%CI)	*P*	OR(95%CI)	*P*
V1	1.000	—	1.000	—	1.000	—
V2	3.203(2.657∼3.862)	<0.001	3.070(2.531∼3.724)	<0.001	1.584(1.205∼2.083)	0.001
V3	7.877(6.585∼9.422)	<0.001	6.977(5.797∼8.398)	<0.001	2.386(1.778∼3.202)	<0.001
V4	20.344(16.972∼24.385)	<0.001	18.668(15.474∼22.523)	<0.001	4.104(2.835∼5.939)	<0.001

Model 1 was not adjusted for confounding factors. Model 2 was adjusted for age and gender. Model 3 was adjusted for the covariates of model 2 plus waist circumference, body mass index, systolic blood pressure, diastolic blood pressure, total cholesterol, triglyceride, low density lipoprotein cholesterol, high density lipoprotein cholesterol, fasting plasma glucose, serum uric acid, serum creatinine, aspartate aminotransferase, alanine aminotransferase, gamma-glutamyltransferasethyroid, thyroid-stimulating hormone. Odds ratios and 95% CIs were calculated per 1-SD increment of VAI. VAI, visceral adiposity index; OR, odds ratio; CI, confidence interval.

### Multivariate logistic regression analysis of the association between FT3/FT4 ratio, VAI and NFS status for NAFLD positive participants

FT3/FT4 ratio and VAI as independent variables were assigned with NFS status as a dependent variable (assignment: high NFS=1, low NFS=0), and multivariate logistic analysis was conducted. The results showed that a negative relationship could be found between FT3/FT4 ratio and the NFS status without adjusting confounding factors 〔OR=0.017, 95%CI (0.005, 0.056)〕 and after adjustment for age and gender 〔OR=0.105, 95%CI (0.026, 0.422)〕 (*P*<0.01) (Model 1 and Model 2). There was no relevance between FT3/FT4 ratio and the status of NFS after further adjustment for WC, BMI, SBP, DBP, TC, TG, LDL-C, HDL-C, FPG, SUA, Scr, AST, ALT, γ-GGT and TSH (*P*>0.05) (Model 3). The results also showed that there was no relevance between the levels of VAI and the NFS status without adjusting confounding factors (*P*>0.05) (Model 1). The VAI was the influencing factor of the NFS status after adjustment for age and gender (*P*<0.01) (Model 2). After further adjustment for WC, BMI, SBP, DBP, TC, TG, LDL-C, HDL-C, FPG, SUA, Scr, AST, ALT, γ-GGT and TSH, there was no significant association between VAI and the status of NFS (*P*>0.05) (Model 3) (**Table 7**).

**Table 7:** Multivariate logistic regression analysis of the association between FT3/FT4 ratio, VAI and NFS status for NAFLD positive participants.

	Model 1		Model 2		Model 3	
	OR (95%*CI*)	*P*	OR (95%*CI*)	*P*	OR (95%*CI*)	*P*
FT3/FT4	0.017 (0.005∼0.056)	<0.001	0.105 (0.026v0.422)	0.001	0.298 (0.043∼2.051)	0.219
VAI	1.021 (0.989∼1.054)	0.207	1.059 (1.021∼1.099)	0.002	0.970 (0.762∼1.236)	0.807

Model 1 was not adjusted for confounding factors. Model 2 was adjusted for age and gender. Model 3 was adjusted for the covariates of model 2 plus waist circumference, body mass index, systolic blood pressure, diastolic blood pressure, total cholesterol, triglyceride, low density lipoprotein cholesterol, high density lipoprotein cholesterol, fasting plasma glucose, serum uric acid, serum creatinine, aspartate aminotransferase, alanine aminotransferase, gamma-glutamyltransferasethyroid, thyroid-stimulating hormone. Odds ratios and 95% CIs were calculated per 1-SD increment of VAI. VAI, visceral adiposity index; OR, odds ratio; CI, confidence interval.

### Mediating effect analysis of visceral adiposity index on the association between FT3/FT4 ratio and the risk of NAFLD

**Figure 2:**
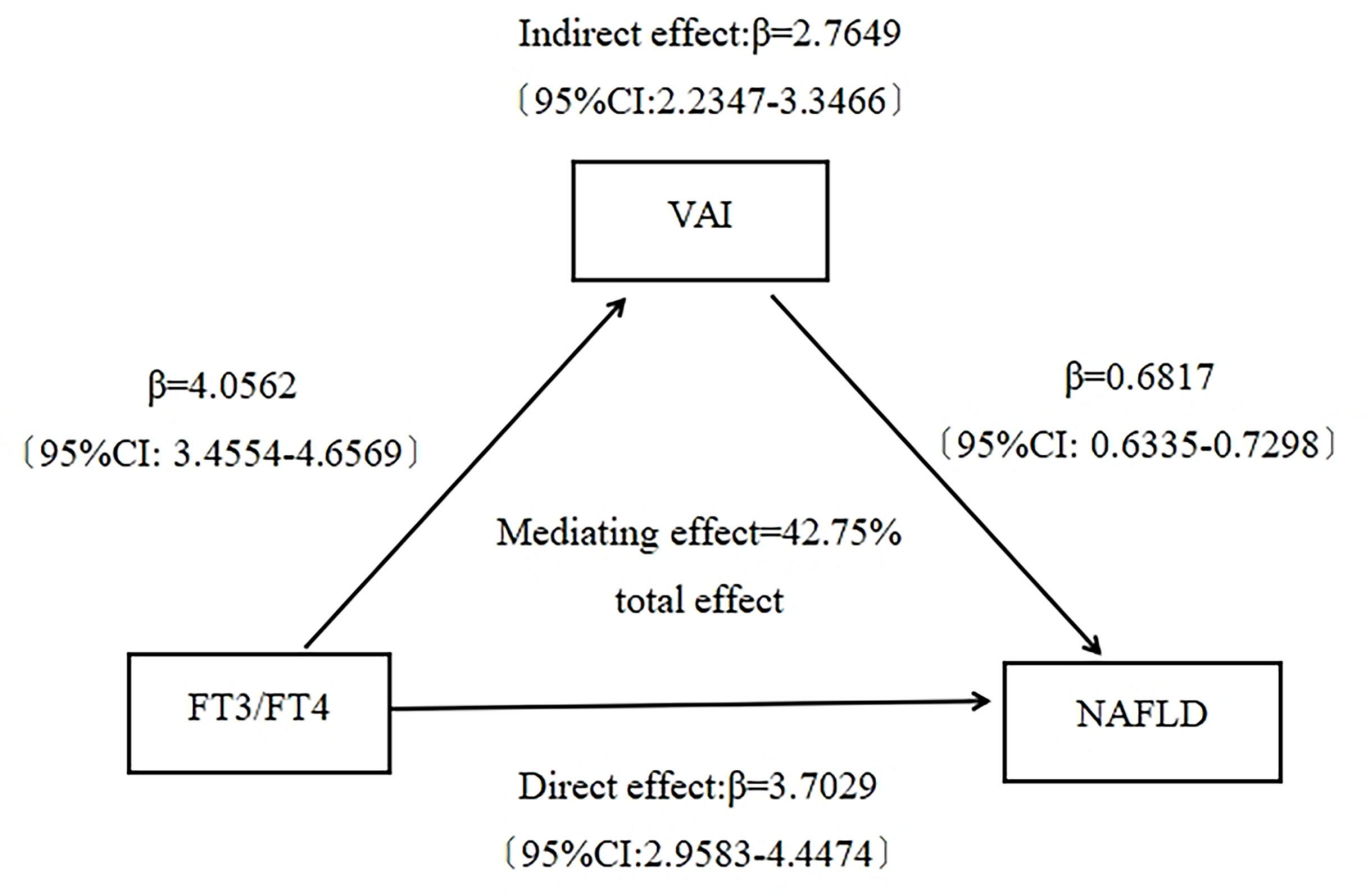
Mediating effect analysis of visceral adiposity index on the association between FT3/FT4 ratio and NAFLD.

This study showed that both FT3/FT4 ratio and VAI were positively associated with NAFLD. Moreover FT3/FT4 ratio was positively associated with VAI. It suggested that VAI could potentially mediate the association between FT3/FT4 ratio and NAFLD. To explore whether VAI mediated the association between FT3/FT4 ratio and NAFLD, we performed a mediation effect analysis using the bootstrap method. The results showed that FT3/FT4 ratio had a significant direct effect on NAFLD risk 〔β=3.7029, 95%CI (2.9583, 4.4474)〕, and VAI partially mediated the indirect effect of FT3/FT4 ratio on NAFLD 〔β=2.7649, 95%CI (2.2347, 3.3466)〕. The mediating effect accounted for 42.75% of the total effect (**Figure 2**).

## Discussion

FT4 can be converted to FT3 by deiodinase, and FT3/FT4 ratio reflects the peripheral sensitivity of thyroid hormones. The present study’s novelty is applying the sensitivity of thyroid hormone indices rather than absolute circulating values of FT3 and FT4 as a predictor of NAFLD risk, which could provide more information on the association between thyroid hormone resistance and NAFLD. This study indicated that in the euthyroid population, the levels of WC, BMI, blood pressure, TC, TG, LDL-C, VAI and NAFLD prevalence increased, whereas the levels of HDL-C decreased with elevated FT3/FT4 ratio. It suggested a higher conversion from fT4 to fT3 due to increased basal metabolic rate and total energy expenditure as a compensatory defense mechanism for fat accumulation to improve energy expenditure (12, 18). In this study, FPG level was no significant difference in groups as FT3/FT4 ratio increased. Multivariate logistic regression analysis showed that FT3/FT4 ratio was positively correlated with NAFLD when no confounding factors were adjusted. After adjusting for multiple confounding factors, a significant positive correlation was observed between NAFLD risk and the FT3/FT4 ratio. It suggested that a higher FT3/FT4 ratio withi7n the euthyroid range was a risk factor for NAFLD prevalence. This complemented clinical evidence for FT3/FT4 assessing NAFLD risk in euthyroid individuals. Roef et al. (19) found that FT3 and FT3/FT4 ratio were positively correlated with BMI, WC, TG, SBP, DBP, FPG and other metabolic syndrome components, but negatively correlated with HDL-C levels, which was basically consistent with the results of this study. The study conducted by Pergola et al. (20) indicated that elevated FT3 could lead to progressive central fat accumulation, which may be an adaptive thermogenesis phenomenon in overweight and obese women with normal thyroid function. At the same time, multiple studies suggested that low FT4 concentrations were closely related to elevated hepatic triglyceride, hepatic steatosis and obesity (21, 22). We found that FT3/FT4 ratio was not a significant influencing factor for the NFS status in the subjects of NAFLD with normal thyroid function. Whereas a single-center study of liver biopsy-proven NAFLD suggested that FT3/FT4 ratio was a valid and valuable predictor of NAFLD, and it was equally influential in assessing the severity of cirrhosis (23). This might be related to different choices of populations.

The underlying mechanism of the association between the FT3/FT4 ratio and NAFLD was not fully understood. The possible mechanism was as follows: elevated serum FT3/FT4 levels can stimulate lipolysis in adipose tissue, thereby increasing the release of free fatty acids, which induces the activation of intrahepatic macrophages and up-regulates autophagy through transport into the liver. It increases triglyceride synthesis in the liver and aggravates hepatocyte damage (10, 24). Furthermore, FT3 is able to increase fatty acid β-oxidation and promote lipid production, which is associated with the occurrence of NAFLD in the liver (25). Elevated FT3/FT4 may also lead to decreased LDL-C receptor expression, which reduces the uptake and breakdown of total cholesterol by hepatocytes, thereby increasing the incidence of NAFLD (18, 26). At the same time, high FT3 levels can regulate mitochondrial uncoupling protein-2 activity in hypothalamic neurons and promote increased appetite (27). Recent studies demonstrated that thyroid hormones regulated the expression of hepatic lipogenic genes; additionally, several genes whose expression changed were also regulated by thyroid hormones in NAFLD (28). What is more, in recent years, some studies have found that thyroid hormone receptor agonists can inhibit hepatic triglyceride synthesis, increase hepatic total cholesterol clearance, reduce lipid deposition, and simultaneously partially enhance insulin sensitivity, promote glucose metabolism, improve inflammation, and have a certain therapeutic effect on NAFLD. Therefore, it has become a therapeutic drug with great potential for treating NAFLD. It also verifies that thyroid hormones play an essential role in the pathogenesis of NAFLD (29, 30).

Currently, visceral adiposity is getting more and more attention. Visceral adiposity index (VAI) is vital for evaluating visceral obesity. VAI level reflects the distribution and accumulation of visceral fat. Studies have indicated that higher visceral fat level was an independent predictor of poor survival in endometrial cancer and an important indicator for evaluating the prognosis of endometrial cancer (31, 32). Visceral adiposity increased the mortality and risk of peritoneal implant metastasis in patients with stage III colorectal cancer (33). Some studies suggested that VAI was also a strong predictor of NAFLD (15). Visceral adipose tissue releases fatty free acid by lipolysis, which flows directly into the portal circulation and becomes the primary source of hepatic triglyceride (34). Eguchi et al. (35) reported that the severity of hepatic fatty infiltration may be affected by visceral fat accumulation in NAFLD patients. Previously domestic and foreign prospective studies have shown that NAFLD risk increased with elevated VAI levels, and it had a tremendous predictive value for the long-term complications of metabolic syndrome and type 2 diabetes (34, 36–38). In this study, VAI was positively correlated with TC, TG, and LDL-C, but negatively correlated with HDL-C, which confirmed the view above. Logistic regression analysis demonstrated that when VAI was in V2, V3 and V4 groups, the risk of NAFLD also significantly increased. There was no correlation between VAI and NFS status in the subjects of NAFLD with normal thyroid function.

Further mediating effect analysis showed that VAI partially mediated the association between FT3/FT4 ratio and NAFLD in the euthyroid population, which indicated that visceral fat accumulation may be the reason for the correlation between FT3/FT4 ratio and NAFLD. It provided a clinical basis for the prevention and treatment of NAFLD. This study suggested that higher FT3/FT4 represented active lipolysis and high plasma FFA and cholesterol. However, the ability of hepatocytes to metabolize FFA and cholesterol did not increase concomitantly in participants with high FT3/FT4 ratio (26). Besides, higher VAI represented more abundant fat storage. And in people with high FT3/FT4 ratio and VAI, lipid molecules from visceral adipose tissue could be continuously transported into the liver, which may lead to NAFLD (15, 24). In addition, insulin resistance and inflammatory markers may also mediate the association between FT3/FT4 ratio and NAFLD (12).

Since this study is a single-center cross-sectional study, the causal relationship between FT3/FT4 and NAFLD cannot be determined. In the future, we need to conduct further multicenter, prospective cohort studies to deeply explore the predictive effect and possible mechanism of FT3/FT4 ratio on NAFLD.

## Conclusion

FT3/FT4 ratio is an independent risk factor for NAFLD, and VAI partially mediates the effect of FT3/FT4 on NAFLD in euthyroid population. Regular monitoring of FT3/FT4 ratio and VAI has a significant clinical significance for preventing and treating NAFLD and related diseases.

## Data availability statement

The raw data supporting the conclusions of this article will be made available by the authors, without undue reservation.

## Ethics statement

The studies involving human participants were reviewed and approved by Ethics Committee of the Hebei General Hospital. The patients/participants provided their written informed consent to participate in this study.

## Author contributions

H-XL and H-JM participated in the study design. H-XL, Y-YR, C-QM and ZL were involved in the conduct of the study and data collection. H-XL, ZL, QN and C-HY made contributions to the data analysis and interpretation of the results. H-XL and H-JM wrote and modified the manuscript and prepared the tables and figures. All authors contributed to the article and approved the submitted version.

## Conflict of interest

The authors declare that the research was conducted in the absence of any commercial or financial relationships that could be construed as a potential conflict of interest.

## Publisher’s note

All claims expressed in this article are solely those of the authors and do not necessarily represent those of their affiliated organizations, or those of the publisher, the editors and the reviewers. Any product that may be evaluated in this article, or claim that may be made by its manufacturer, is not guaranteed or endorsed by the publisher.
